# Contribution of the mix renewable energy potentials in delivering parts of the electric energy needs in the west region of Cameroon

**DOI:** 10.1016/j.heliyon.2023.e14554

**Published:** 2023-03-15

**Authors:** Nasse Fetio Ngoune, Boris Merlain Kanouo Djousse, Grisseur Henri Djoukeng, Cherelle Grace F. Nguimeya, Kewir Julius Tangka, Martin Tchoffo

**Affiliations:** a Laboratory of Renewable Energies, Department of Rural Engineering, Faculty of Agronomy and Agricultural Sciences of the University of Dschang, 222 BP, Dschang, Cameroon; b Department of Physics, Faculty of Sciences of the University of Dschang, 67 BP, Dschang, Cameroon; c Centre d’Études et de Recherches en Agronomie et Biodiversité (CERAB), Faculty of Agronomy and Agricultural Sciences of the University of Dschang, 222 BP Dschang, Cameroon

**Keywords:** Biomass, Solar energy, Hydroelectricity, Sustainable energy, Spatial distribution

## Abstract

The constant supply of energy remains a great challenge in many developing countries and Cameroon is no exception. It is necessary to explore other renewable energy sources that have environmental and energy potential. However, there is limited and sparse literature on the potential of renewable energy sources in Cameroon and its western part in particular. This limits investment and policy design that can lead to the exploitation of renewable energy sources. There is thus a need for more research on renewable energy development to better inform energy policies. This paper investigates the potential and extent to which available renewable energy sources can contribute to the electric power sector in the western part of Cameroon is on estimating the potential of hydroelectric, solar and biomass energy resources. A cross-sectional method, observations and literature review were used to determine the water flow and electrical energy potential of different biomass. The results show that the electrical potential of hydroelectricity is 11.68 GWh/year, for solar represents 44.12 GWh/year, and the energy of biomass 8586.42 GWh/year, 135.53 GWh/year and 13.05 GWh/year for agricultural, animal and forestry residues; they have a rate of access to electricity of 6.64%, 25.08%, 4881.46%, 77.05%, and 7.42% respectively. This potential can satisfy needs of 18 526 464 households. According to results obtained and in order to provide a sustainable solution by improving access to electricity, living standards and socio-economic conditions of populations; two hybrid cogeneration thermal-solar power plants can be installed at the limits of the decentralized areas of Bamboutos-Mifi-Menoua and Noun-Koung Khi, which are nearby areas with high population density. Hydroelectric plants can be installed to electrify villages that are far from the national network.

## Highlights

• Potential of exploitable renewable energy sources in the western part of Cameroon.
• Number of households to be satisfied by renewable energy sources.
• Improving the rate of access to electrical energy using biomass.
• Informing energy policies on the profitability of investments in the energy sector.

## 1 Introduction

Energy is used at several scales to satisfy the daily needs of human beings and develop the economy of a country [1]. This energy is not always available, worldwide, about 1.2 billion people do not have access to electricity, mostly in rural areas [2]. The greatest difficulties are encountered in the African continent, where access to electricity in rural areas is barely 10–15% in some countries [3]; this poses a significant threat to the economy and well-being of people. Aware of the stakes involved in this strategic resource, states and governments have set up policies that promote the development of the economic sector and political stability [4]. Renewable energies are part of this logic, as they are at the heart of the issue of economic development, the fight against poverty and the reduction of greenhouse gases [5]. Cameroon has real unmet energy needs that contrast with the diversity of renewable energy sources throughout the country. Yet it has immense renewable energy resources that are varied and randomly distributed throughout the country [5]. Bioenergy can satisfy several times the current and future energy demand, their potential is enormous [6]. In this country, hydroelectricity is the most widely used energy. Cameroon has the second largest hydroelectric potential in Sub-Saharan Africa. Despite this, it faces an imbalance between an unstable supply and a growing demand for electricity due to the low water period [7]. This potential is currently estimated at over 115 billion kWh/year. The installed hydroelectric power generation capacity is 723 MW, including the Song Loulou (384 MW) and Edéa (267 MW) dams on the Sanaga River, which account for 97% of the country’s hydroelectric power generation [8], and the Lagdo Dam (72 MW) on the Benue River [7]. With regard to solar energy, Cameroon has an abundant and available potential as it is found in the tropics, but very little exploited. According to the latest studies, wood energy, remains the primary energy vector in rural and urban areas in Cameroon. In 2010, it accounted for 72.60% of total energy consumption, against 20.10% for oil and gas products and 7.30% for electricity [7]. The rate of access to electricity has increased significantly at the national level. It increased from 29% in 1990 to 54% in 2012 and represents an access rate that is about double that of the countries in the Economic Community of Central African States (ECCAS), of which Cameroon is a member [9]. However, the electricity access rate is estimated at 96% and 35% in 2014 in urban and rural areas, respectively. This rate does not reflect the strong disparities with respect to habitat, urban and rural, and geographic regions [9]. Yet that the exploitable hydraulic potential for electricity generation in the areas is quite considerable. However, the West region, like other regions of Cameroon, frequently suffers from a low rate of electricity supply (62.95%) and poor quality of electrical service [10]. It results in voltage drops and load shedding that damage electrical appliances and household appliances, prevents the smooth running of socio-economic activities and significantly affects the quality of life of the population [11]. In large cities, this lasts four to 6 h, and in rural areas, people can be plunged into darkness for three to four days. This is a real ordeal, with increasingly disastrous social and economic consequences [7]. The difficulties in supplying electricity lead to losses estimated at more than US$99 million, or more than one point of the country’s annual growth rate. Industries, large consumers of electricity, are the most affected [7]. Cameroon’s energy balance sheet, however, shows a clear dominance of untapped renewable energy, and especially a marked dependence on biomass, estimated at 81.20%, used for cooking and household heating. It is followed by 15.40% for oil and 3.40% for hydroelectric power in the country’s energy supply [8]. According to Nguesseu et al. [5], renewable energy in Cameroon represents 25% of the electricity mix and is expected to contribute to a 32% reduction in greenhouse gas emissions by 2035. In this context, bioenergy produced from forests is of strategic importance to meet the growing energy demand [12]. Yet the country is endowed with many untapped renewable resources, including solar, hydro, biomass and wind. Added to this, there is almost no research that presents the different renewable energy sources and their potentialities in the West Cameroon locality. This limits investments and the design of energy policies that can lead to the exploitation of the different renewable energy sources. The mastery and valorization of this potential will have a significant impact and will constitute a real opportunity for local development and the increase of energy supply within the framework of the regional electricity mix. Added to this, decentralization is a determining factor that will allow the development of the renewable energy sector in each locality [5,13]. With Law No. 2004/017 of July 22, 2004, on the orientation of decentralization, the State of Cameroon has transferred certain competencies with the appropriate means for their implementation. The energy sector is one of these competencies, including decentralized rural electrification for rural areas that cannot be connected to the national grid. For an efficient exploitation of these resources, it is necessary to intensify research on the development of renewable energies to better inform energy policies. The main objective of this study is to contribute to a better control and valorization of the different renewable energy sources, to simultaneously meet the electricity demand in the West Cameroon region. Better still, to reduce the level of poverty of the populations. More specifically, it will assess and map the energy potential, hydroelectric, solar, plant biomass, forest biomass and animal biomass in West Cameroon.

## 2 Materials and methods

### 2.1 Electricity potential of hydroelectric plants

To evaluate the hydroelectric potential of the region, the first step was to select potential rivers from which energy could be harnessed. This choice was based on data from previous studies conducted 19 years ago by Tekounegning [14], and on interviews with resource persons such as riparian, village chiefs and museum representatives. Streams with at least 2 m falls, and sufficient flow were considered exploitable [15]. The median cross-section method was used to estimate the flow of each stream during low flow periods (late February and early March). The number of verticals was placed according to the width of the river, as recommended by Niyonzima and Hendrick, [16]. The current meter of brand name QUALIMETRICS with the accuracy of 0.01 was used to measure the velocity of each subsection. Eq. (1) proposed by Greg [17], was used to calculate the partial flows. A total of 48 waterfall points located at 21 villages in the western region were explored.

(1) $$q_{i}=v_{i}\left[\frac{\left(L_{i}-L_{i-1}\right)}{2}+\frac{\left(L_{i+1}-L_{i}\right)}{2}\right]p_{i}=v_{i}\left[\frac{\left(L_{i+1}-L_{i-1}\right)}{2}\right]p_{i}$$

where: $q_{i}$ = partial flow rate passing through subsection (panel) i, in m^3^/s; $v_{i}$ = average speed on the vertical i, in m/s; $L_{i}$ = distance from the initial point to the vertical i in m and $p_{i}$ = depth of water at vertical I, in m.

Eq. (2) [17] was used to calculate the total streamflow. This flow is the sum of the partial flows corresponding to each vertical.

(2) $$Q=q_{1}+q_{2}+q_{3}+\ldots q_{n-1}+q_{n}$$

where: Q = total flow in m^3^/s; $q_{1}+q_{2}+q_{3}+\ldots q_{n-1}+q_{n}$; $q_{i}$ partial flow of each subsection in m^3^/s.

**Fig. 1:**
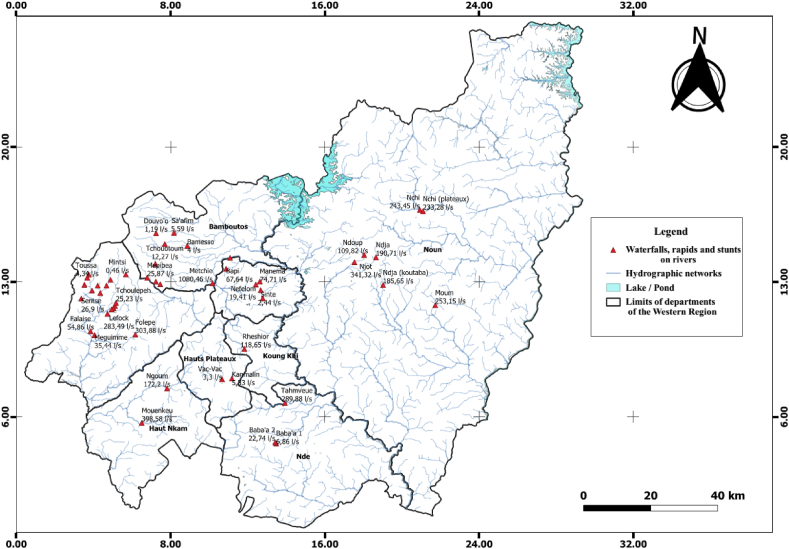
Representation of rivers with falls, stunts and rapids explored in the West of Cameroon region.

A GPS of brand name GARMIN (GPSMAP 64) was used to record the upstream (Z1) and downstream (Z2) elevations of the explored drop points. QGIS version 3.18 software and the geographic coordinates of each drop point were used to produce a georeferenced map (Fig. 1) of the drop points, rapids, and cascades.

The hydroelectric energy potential was calculated using Eq. (3) [18]. The efficiency of hydroelectric equipment ranges from 80 to 90% [18]. For this study, the overall efficiency of 80% is used to determine the electrical output. This ratio takes into account the efficiency losses of the turbine, generator and transmission parts [15].

(3) $$P_{el}=0,80\times g\times Q\times K\times T$$

where: $P_{el}$ = electrical energy at the output of the alternator in kWh; Q = stream flows in m^3^/s; K = gross head of water in m, T = time of operation of the plant per day estimated at 24 h and the normal value of acceleration of the terrestrial gravity, noted g, equal to 9,81 m/s^2^.

The number of households (Eq. (4)) that could benefit from the energy generated by small hydropower plants was estimated based on the average electricity consumption estimated at 39 kWh/month or 1.3 kWh/day. This consumption is limited to lighting and basic services with low average power appliances, and to some extent refrigeration in the rural area [9].

(4) $$N_{n}=\frac{P_{el}}{B_{moy}}$$

where: $N_{n}$ = current number of households and $B_{moy}$ = average energy needs in kWh/day.

### 2.2 Potential of solar electrical energy

#### 2.2.1 Global solar irradiation

To estimate the solar energy potential, the empirical model equations of Liu and Jordan below were used; with the advantage of this model being that it can generate the solar flux received at the ground for different sky states and surface inclinations [19]. The global irradiance was calculated using the Matlab code developed with these equations. The height of the sun, the angle formed by the direction of the sun and its projection on the horizontal plane were determined to use equation (5) [20].

(5) sinh=cos(δ)×cos(φ)×cos⁡(ω)+sin(φ)×sin(δ)

where: φ = latitude of the location in degree; δ = declination of the sun and ω = hourly angle in degree.

Eq. (6) [21] was used to determine the solar declination (δ) which is the angle in between the direction of the sun and the equator.

(6) $$\delta =23,45.\sin\left(\frac{2.\pi .\left(N+284\right)}{365}\right)$$

where: N is the number of days of the year, varying from 1 to 365. (On January 1, N = 1 and December 31, N = 365 days).

Eq. (7) [20] allowed us to determine the hourly angle. It is the angle between the meridian of origin passing through the south and the projection of the direction of the sun on the equatorial plan.

(7) ω=15(12−TSV)

where: $TSV=H+\left(\frac{\gamma }{15}\right)+E^{t}$ ; TSV = true solar time in hours; H = time GMT; λ = longitude East of the place in degree [E] and $E^{t}$ is the equation of time which is expressed by the following relation. With

$$E^{t}=\left(9,87\times \sin\left(2B\right)\;–\;7,5\times cosB\;–\;1,5\times \sin\;B\right)\times \frac{1}{60}and\;B=\frac{2\pi \times \left(N-81\right)}{364}$$

Eq. (8) [20] was used to calculate the direct solar radiation I which passes through the atmosphere without undergoing any modification on a horizontal plan.

(8) $$I=\mathrm{Asin}\left(h\right)\exp\left(\frac{-1}{Csin\left(h+2\right)}\right)$$

On the horizontal plan, β=0 so $R_{b}=1$. For a horizontal plane, the reflected solar component is zero [22]. The values of A, B and C are constants that take into account the nature of the sky [20].

The diffuse solar radiation on a horizontal plan was calculated using Eq. (9) [21]. Diffused radiation is the portion of solar radiation scattered by solid or liquid particles suspended in the atmosphere.

(9) $$D=B(\sin{\left(h)\right)}^{0,4}$$

where: h = angle formed by the horizontal plane of the location considered and the direction local point – Sun.

Eq. (10) [20] was used to determine the global radiation which is the sum of the direct and diffuse solar components. The average insolation duration used to estimate is 6.27 h/day [23].

(10) $$G=\left(I+D\right)\times P_{K}$$

where: G = global irradiation in W/m^2^; D = diffuse irradiation in W/m^2^; I = direct solar radiation in W/m^2^ and $P_{K}$ = average insolation time.

#### 2.2.2 Theoretical area of photovoltaic (PV) modules

The theoretical area of PV modules depends on the electricity consumption and the solar resource available in each country. It is expressed as a % of the country’s area [24]. The total consumption requirement needed to fill the electricity gap in the region was used in this study.

Eq. (11) [24] was used to determine the solar electricity potential that could be produced daily on a horizontally placed photovoltaic (PV) system in a county.

(11) $$E=P_{R}\times G\times P_{s}\times S_{t}$$

where: E = electrical energy generated per day by a photovoltaic system kWh; G = annual or monthly global irradiation on the horizontal plane in kWh/m^2^; $S_{t}$ = theoretical area of the field in %; $P_{R}$ = efficiency of the solar panel estimated at 18% [25] for mono, multi and polycrystalline silicon modules) and $P_{s}$ = performance ratio of the systems, it is equal to 0.75 [24].

### 2.3 Energy potential from crop, forest and animal biomass

Biomass data were obtained from local administrative departments of agriculture, forestry, wildlife and livestock. The recoverable fraction of agricultural biomass residues generated during harvest that could realistically be exploited was estimated to be 70% of the total amount available [26]. This percentage allows for a fraction of residues to be left in the field to maintain agricultural soil quality and soil erosion control [27]. Eq. (12) [6] was used to estimate the energy potential of crop residues using the data obtained from year 2018 to year 2020. Parameters such as calorific value and residue/product ratio of the different crops were obtained from the available literature. This method is also applied to the calorific values of biomass residues and biogas generated by the manure of different animal species. Appendix 1 presents the parameters used for each type of agricultural residue in this work.

(12) $$Q_{AR}=\sum_{i=1}^{n}\left(C_{i}\times RPR_{i}\times \left(SAF+EUF\right)\times r\times LHV_{i}\right)$$

where: $Q_{AR}$ = annual gross energy potential of agricultural residues in PJ; $C_{i}$ = annual production of crops i in kg; n = total number of residue categories; $RPR_{i}$ = residue to product a ratio of crops i; $LHV_{i}$ = lower heating value of a given crop residue in MJ/kg; SAF = dimensionless surplus availability factor; EUF = energy utilization factor and r = recoverable fraction.

Wood chips and charcoal are used for household heating and cooking and for electricity generation. Eq. (13) proposed by Smeets and Faaij [28] was used to calculate the energy potential of forest residues that can be recovered. This calculation was done using the data obtained from the years 2019–2021. The lower heating value of the firewood and charcoal used was 25 MJ/kg, as recommended by Crehay and Marchal [29].

(13) $$Q_{HR}=\sum_{i=1}^{n}\left(W_{i}\times h\times F\times LHV_{i}\right)$$

where: $Q_{HR}$ = energy potential of fuel wood in MJ; $W_{i}$ = annual firewood production in kg and $LHV_{i}$ = lower heating value of fuel wood in MJ/kg.

The logging residue generation factor (h) and the logging residue recovery fraction [F] vary according to the type of residue [28]. According to Yamamoto et al. [30] both factors are equal to 1 for fuel wood in developing countries. The energy potential of the generated wood processing residues was estimated using Eq. (14) [6].

(14) $$Q_{HR}=\sum_{i=1}^{n}\left(W_{IR}\times p\times P\times LHV_{h}\right)$$

where: $Q_{HR}$ = energy potential of charcoal in MJ; $W_{IR}$ = annual charcoal production in kg and $LHV_{h}$ = lower heating value of charcoal in MJ/Kg.

The rate of generations of processing residues [p] and the recoverable fraction of these residues [P] for developing countries are 1 each for wood, and 0.15 and 1, respectively for charcoal [31].

The energy potential of animal residues produced by cattle, sheep, goats, pigs and poultry was considered. Indeed, these are the most raised species in the western region. According to Okello [32], the daily production of volatile solids per animal and the biogas yield per kilogram of volatile solids are the properties of animal manure needed to estimate its energy potential. These parameters were obtained from the literature and used to estimate the amount of biogas that can be produced by each category of animal species. Eq. (15) [33] was used to calculate the amount of biogas that can be produced by livestock category, along with these different parameters used. The energy potential was calculated using the data obtained from the years 2019–2021.

(15) $${EP}_{A}=365\times N_{h}\times Dm\times F_{r}\times V_{s}\times B_{y}\times {LHV}_{Biogaz}$$

where: ${EP}_{A}$ = recoverable biogas energy potential in MJ/J; $N_{h}$ = number of animals per head; Dm = amount of dry matter per head in kg/day; $F_{r}$ = recoverable mass fraction; $V_{s}$ = volatile matter which is the volatile part of the organic matter, i.e. the mass fraction of the Dm [%]; $B_{y}$ = biogas yield over $V_{s}$ in m^3^/kg and ${LHV}_{Biogaz}$ = lower heating value of biogas 20 MJ/m^3^ and 365 is the number of days per year.

### 2.4 Spatial distribution of the electrical energy potential of renewable resources

QGIS version 3.18 software was used to produce the spatial distribution map of the electrical energy that could be produced by hydraulic, solar and biomass resources in the West Cameroon region.

The electricity access rate represents the proportion of households that could be electrified [34]. It is determined for each energy source by dividing the number of electrifiable households per department by the total number of households in the West Cameroon region. This indicator is more relevant than the coverage rate, which does not give the actual population connected, but the population covered in electrified regions [9].

### 2.5 Ethics statement

An ethics statement is not required for our study, since no investigations were done on human beings.

## 3 Results

### 3.1 Electrical energy potential of hydro, solar and biomass

#### 3.1.1 Energy from hydroelectric power plants

Appendix 2 presents the heights, flows, and hydroelectric power of the rivers explored in the West Cameroon region. Appendix 2 shows that the Noun department has waterfalls with high flows at the end of the dry season. The low flow rate, less than 5 l/s in some of the rivers explored, is due to the withdrawal of water by local residents for the development of their activity.

**Fig. 2:**
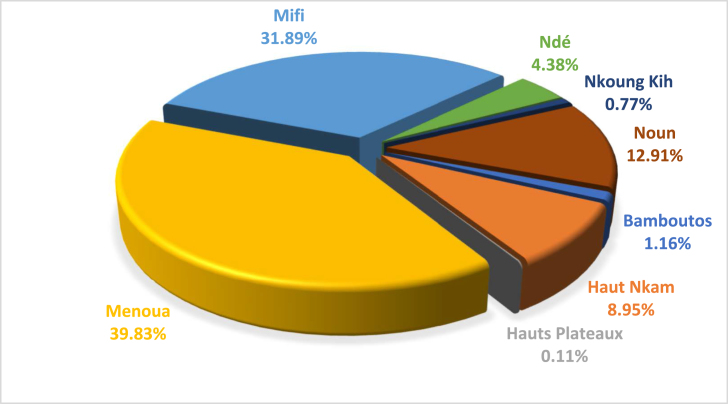
Proportion of hydroelectric power obtained by department in the western region.

The height of the falls and cascades varies from 2.06 m to 110 m. Menoua has more waterfalls than the other departments. This parameter, together with the flow rate, has resulted in a total theoretical hydroelectric power of 32004.85 kWh/day. This will satisfy the daily electrical energy needs of 24 619 households. Fig. 2 shows the proportions of hydroelectric energy obtained for each department of the western region.

The most important theoretical hydroelectric energy is obtained in Menoua with 12,747.47 kWh/day representing 39.83% of the total potential obtained in the region. It would allow for the satisfaction of approximately, 9806 households. It is followed by Mifi and Noun, which respectively have a theoretical energy of 10,206.46 kWh/day and 4131.67 kWh/day with proportions of 31.89% and 12.91%. Their electrical energy would supply, 7851 and 3178 households. The Hauts Plateaux, Ndé, Koung Khi, Haut Nkam and Bamboutos have a low level of hydroelectric energy (4919.25 kWh/day). This represents 15.37% of the hydroelectric power potential obtained in the said region.

#### 3.1.2 Electrical solar energy

**Fig. 3:**
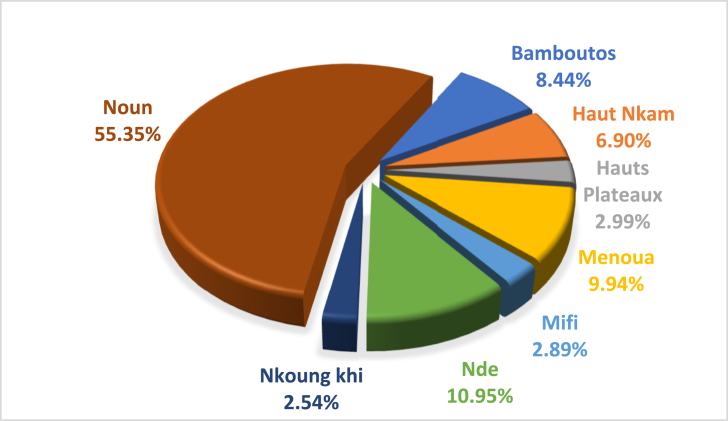
Percentage of solar potential for electricity production in the West Cameroon region.

The overall average annual horizontal irradiation in the western locality is 2388.43 kWh/m^2^/year. It is almost constant by department and varies between 2387.08 kWh/m^2^/year and 2389.58 kWh/m^2^/year. The average annual potential of nominal electrical energy production at the horizontal is 322.44 kWh/m^2^/year. The total electrical potential received on the theoretical surface of the western region is about 120,889.67 kWh per day. It is obtained with a theoretical surface estimated at 0.0009% of the total area of each department of the Western Region. This potential will allow the electrification of approximately, 92,992 households per day in West Cameroon. Fig. 3 presents the percentages of nominal electrical potential by department in the West Cameroon region.

Fig. 3 shows that the nominal electrical potential is high in Noun with 67,902.98 kWh/day, representing 55.34% of the total potential of the Western Region due to its larger area. It is followed by Ndé (13,237.33 kWh/day) and Menoua (12,011.34 kWh/day). This different electrical potential will make it possible to satisfy the daily electrical needs of 51,468; 10,183 and 9239 households respectively. The lowest nominal solar electric potential is obtained in Nkoung kih with 3073.00 kWh/day representing 4.59% of the Western Region’s potential. It will satisfy 2364 households daily in this department.

#### 3.1.3 Electrical energy from biomass

**Fig. 4:**
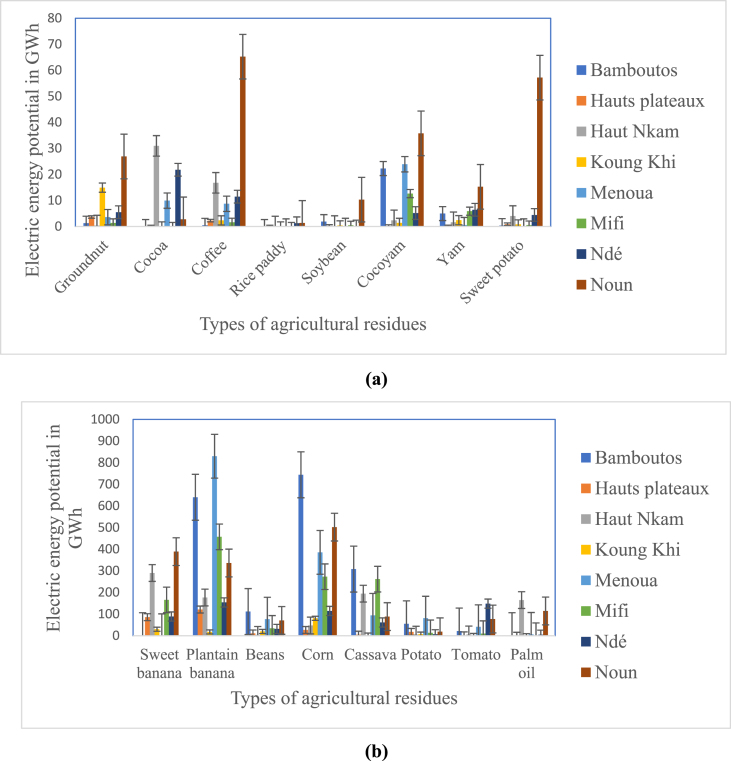
Theoretical electric energy potential of agricultural residues, (a) varies from 0 to 70 GWh/year and (b) from 70 to 1000 GWh/year in the West Cameroon region.

Fig. 4a and b present the electrical energy potential of the agricultural residues studied in this work. The analysis shows that crops such as plantain, maize, bananas and cassava have the highest theoretical electrical energy potential per department estimated at 2732.07 GWh/year; 2173.17 GWh/year; 1048.70 GWh/year and 1015.48 GWh/year respectively in the West region. However, Noun, Bamboutos and Menoua have the highest potential for electrical energy. The lowest is obtained in the department of Koung Khi. The lowest electrical energy by department is observed in bean, tomatoes, oil palm and potato residues with respectively an energy potential of 358.25 GWh/year, 306.91 GWh/year, 290.19 GWh/year and 204.59 GWh/year. Taro, rice, soybeans, sweet potatoes, yams, coffee, cocoa and groundnuts have low potential in all departments. Total energy from these different crops is estimated at 457.07 GWh/year in the West Region. The energy produced by rice biomass is even lower than the others because it is only produced in two departments; Ndé and Noun.

**Fig. 5:**
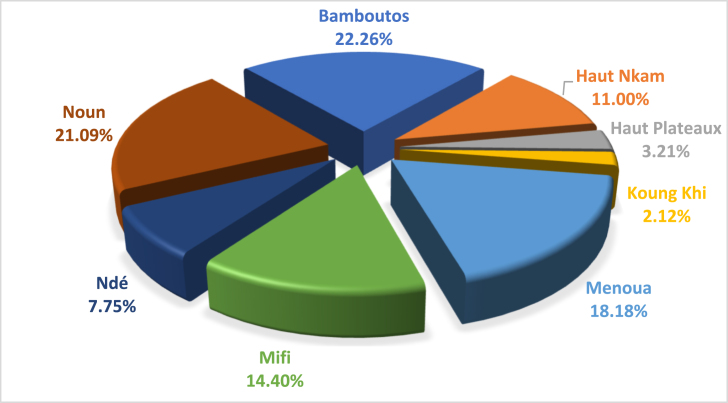
Electricity production rate of agricultural residues by department in the West Cameroon region.

The theoretical electrical energy potential of agricultural residues is about 8586.42 GWh/year and represents 23.52 GWh per day. Fig. 5 shows that the electrical energy potential of agricultural residues is high in Bamboutos (22.26%) because of customs and habits. It is followed by Noun, which represents 21.09% in the West. The department of Hauts Plateaux and Koung Khi record the lowest percentages of electrical energy production, with 3.21% and 2.12% respectively.

**Fig. 6:**
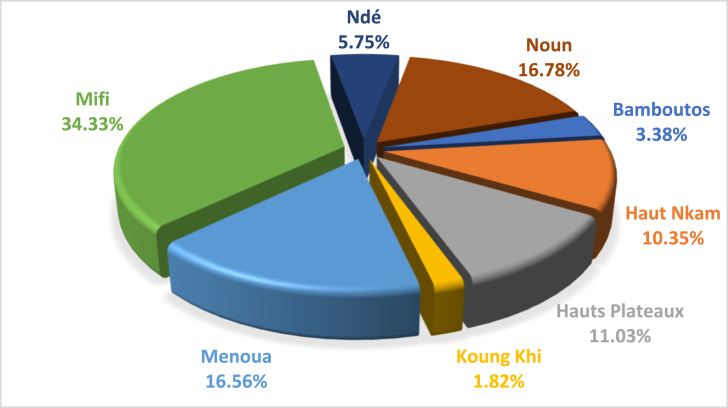
Rate of electrical energy generation from forest residues in the West Cameroon region.

The total potential for theoretical forest electrical energy is estimated at 13.05 GWh/year, 95% of which is dedicated to firewood and 5% to charcoal in the Western Region. However, firewood is used more in the Mifi, Noun, Menoua, Hauts Plateaux and Haut Nkam departments. This is because they have a theoretical electrical energy potential estimated at 4.32 ± 1.67 GWh/year; 2.06 ± 1.80 GWh/year; 1.88 ± 1.62 GWh/year; 1.44 ± 0.10 GWh/year and 1.35 ± 0.54 GWh/year respectively. Ndé, Koung Khi and Bamboutos have the lowest potential, with a total of 1.40 GWh/year. As for charcoal, Menoua has the highest electrical potential 0.28 ± 0.17 GWh/year. Analysis of Fig. 6 shows that the percentages of forestry electrical energy are high in Mifi Department 34.33% or 4.48 GWh/year and Noun 16.78% representing 2.19 GWh/year; it is roughly equal to that of Menoua which has 16.56% or 2.16 GWh/year. The lowest potential is observed in Bamboutos, Koung Khi with 3.38% and 1.82% representing 0.44 GWh/year and 0.24 GWh/year of forestry electricity, respectively.

**Fig. 7:**
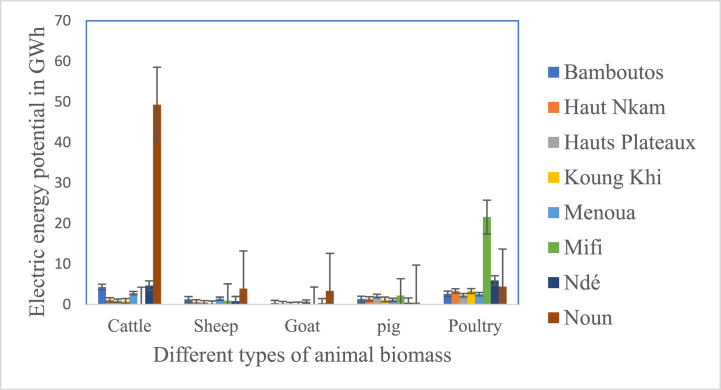
Electrical energy potential of animal manure.

Analysis of Fig. 7 shows that the theoretical electrical potential generated by cattle dung is the highest and represents 49.28 ± 0.14 GWh/year in Noun. However, Ndé, Bamboutos and Menoua have a potential of 4.64 ± 0.60 GWh/year; 4.32 ± 0.12 GWh/year and 2.81 ± 0.03 GWh/year, respectively. The other departments have a low total estimated electrical potential of 3.08 GWh/year. Poultry manure in Mifi has the highest electricity potential (21.59 ± 18.35 GWh/yr) compared to the other departments. It is followed by Ndé and Noun, which respectively have an electrical energy of 5.91 ± 9.64 GWh/year and 4.42 ± 3.63 GWh/year. The other departments have a total electrical potential of 14.15 GWh/year. The electrical potential generated by the waste of other species has a total of less than 4 GWh/year per department.

**Fig. 8:**
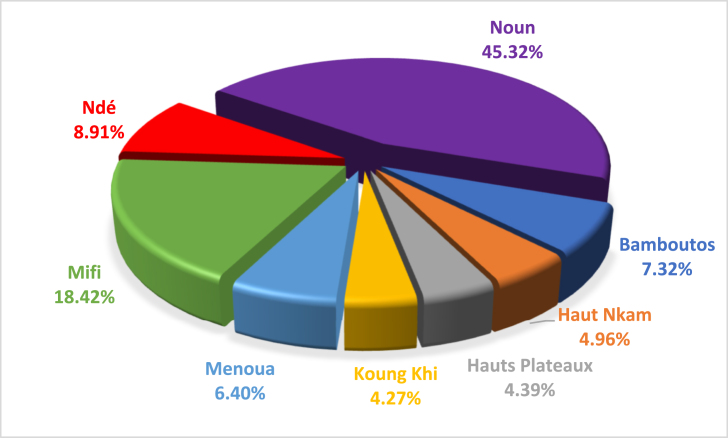
Percentage of electrical energy from animal manure in the West Region of Cameroon.

Analysis of Fig. 8 shows that the percentages of electrical energy generated by animal waste are high in Noun Department 45.32% representing 61.43 GWh/year and medium in Mifi, with 18.42% and worth 24.96 GWh/year. The lowest potential is observed in Koung Khi with 4.27% which is equivalent to 5.79 GWh/year.

### 3.2 Summary of the electrical potential of energy sources

Table 1 presents a summary of the electrical potential of some exploitable energy sources of about 8790.81 GWh/year in the West Cameroon region. Nevertheless, the electrical potential produced by agricultural residues is the highest (8586.42 GWh/year) and represents 97.67% compared to other sources. This potential is medium with animal manure; low with hydroelectric potential and represents 135.53 GWh/year, 11.68 GWh/year and is equivalent to 1.54% and 0.13%, respectively.

**Table 1:** Estimation of the theoretical electrical potential of some energy sources in West Cameroon.

Localities of the west region	Hydroelectricity (GWh/an)	Solar energy (GWh)	Animal residue (GWh/an)	Agricultural residue (GWh/an)	Forest residue (GWh/an)	Total electrical energy (GWh/an)
Bamboutos	0,14	3,72	9,93	1911,48	0,44	1925,71
Haut Nkam	1,05	3,05	6,72	944,37	1,35	956,53
Hauts Plateaux	0,01	1,32	5,95	275,34	1,44	284,06
Koung Khi	0,090	1,12	5,79	181,63	0,24	188,87
Menoua	4,65	4,38	8,67	1561,21	2,16	1581,08
Mifi	3,73	1,28	24,96	1236,41	4,48	1270,85
Ndé	0,51	4,83	12,08	665,36	0,75	683,53
Noun	1,51	24,42	61,43	1810,62	2,19	1900,17
Total	11,68	44,12	135,53	8586,42	13,05	8790,81

The results of the sum of the sources of electrical energy by department are high in Bamboutos and Noun, estimated at 1925.70 GWh/year (21.91%) and 1900.16 GWh/year (21.62%). The lowest potential is obtained in the Haut Plateaux and Koung Khi with 284.06 GWh/year and 188.87 GWh/year which represents 3.23% and 2.15%, respectively of the total potential produced in the Western region.

### 3.3 Number of households to be electrified by the source of electrical energy

This energy is capable of meeting the electrical energy needs of 18,526,464 households in the Western Region. Table 2 shows that the electrical potential of agricultural residues can satisfy more households than other sources. The greater the electrical potential, the greater the number of households to be satisfied with electrical energy.

**Table 2:** Number of households to be electrified daily by electrical energy sources in the West Cameroon region.

Localities of the west region	Hydroelectricity	Solar energy	Animal residue	Agricultural residue	Forest residue	Total households
Bamboutos	286	7850	20 920	4 028 409	929	4 058 394
Haut Nkam	2204	6418	14 172	1 990 233	2846	2 015 873
Hauts Plateaux	26	2779	12 546	580 274	3034	598 659
Koung Khi	189	2364	12 206	382 790	501	398 050
Menoua	9806	9239	18 275	3 290 225	4554	3 332 099
Mifi	7851	2691	52 608	2 605 703	9440	2 678 294
Ndé	1079	10 183	25 453	1 402 233	1581	1 440 529
Noun	3178	51 468	129 457	3 815 848	4615	4 004 565
Total households	24 619	92 992	285 637	18 095 715	27 501	18 526 464

**Fig. 9:**
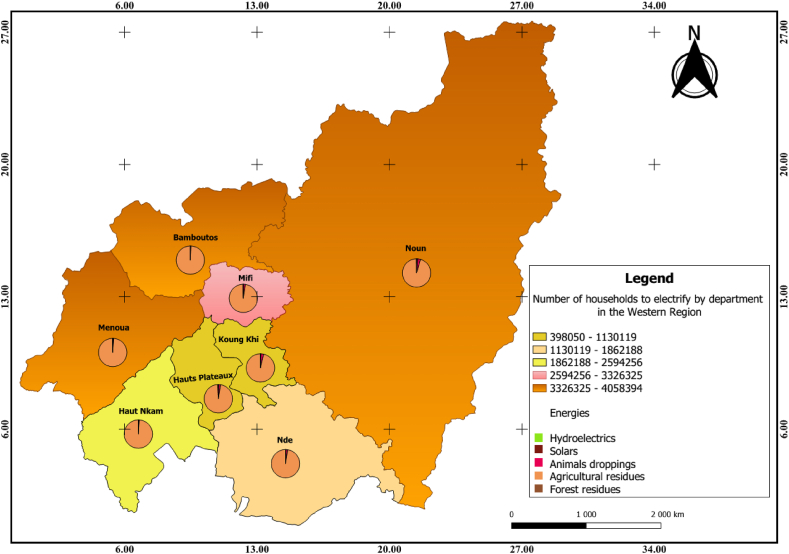
Distribution of the number of households to be electrified and the electricity potential of the different energy sources in the Western Region.

The spatial distribution of the number of households (Fig. 9) on the map of the West Cameroon region shows that Bamboutos, Noun and Menoua are the departments with the highest potential number of households in the region. However, the pie chart shows that the greatest electrical potential is observed in agricultural residues. Other energy sources are poorly represented.

### 3.4 Electricity access rates by renewable energy source

Table 3 shows that access to electricity will be high with electrical energy produced from agricultural residues, which has a percentage of about 4881.46%. This proves that this source of energy can improve present and future electricity needs in the West Cameroon region. The access rate to electricity generated from animal dung is average and can satisfy the electricity needs of 77.05% of households in the West region. Electricity generated from forest residues and hydroelectricity can only meet the needs of 7.42% and 6.64% of households, respectively in the West Cameroon region.

**Table 3:** Rate of access to electricity by energy sources in the West Cameroon region.

Source of electrical energy	Hydroelectricity	Solar energy	Animal manure	Agricultural residues	Forest residues
Rate of access to electricity in %	6,64	25,08	77,05	4881,46	7,42

## 4 Discussions

### 4.1 Hydroelectricity potential of the West Cameroon region

The variation in river flows measured in 2002 and 2022 is due to climatic variability and the development of activities along the measured streams. Farmers using these streams use gravity irrigation with traditional sprinklers for more than 80% (flows varying from 0.067 l/s to 0.10 l/s) to irrigate their market garden produce for a period of three to four months. In addition, the mountainous terrain of the western region is also conducive to the use of runoff water volumes [35]. All of the streams surveyed have an average head of 34.06 m and flows of 143.85 l/s. The exploitable electrical potential of the streams is 32,004.85 kWh/day. It will enable satisfying the daily low-voltage consumption of about 24 619 households. This represents an electricity access rate of 6.64% in the Western Region. This rate is low compared to the Western Region 66.6% [9]. A global study estimated the exploitable potential of hydro plants in Cameroon at 1.115 TWh. The Western (35%) and Eastern (34%) regions have the most interesting application perspective [36]. The work of Tekounegning [14], shows that the hydroelectric potential in the West Cameroon region are high head and low-flow developments, which corroborates our results. Pelton turbines are therefore better suited for their development.

The existence of several permanent streams with falls and rapids is an asset for the development of the West Cameroon region. The government should take advantage of and develop pico (≤20 kW) and micro (20 kW–500 kW) hydroelectric power plants with check dams [37] to provide electrical energy to villages far from the national electricity grid. In order to boost the standard of living of the populations living in the villages.

### 4.2 Electrical solar energy in the West Cameroon region

The annual horizontal global irradiation in the western locality is almost constant and varies between 2387.08 kWh/m^2^/year and 2389.58 kWh/m^2^/year with an average electricity production of 0.88 kWh/day, which is 322.44 kWh/m²/year. Ngoya et al. [38], had obtained a global irradiation potential ranging from 1970 kWh/m^2^ to 2231 kWh/m^2^ in the West Cameroon region using the PV GIS approach. The difference observed between these data is due to the nature of the sky and the average duration of insolation in the area, as it impacts the solar potential that is received at a location. The solar energy produced by the department varies between 3073.00 kWh/day and, 66,908.56 kWh/day. It would theoretically satisfy the electricity consumption of households in the West Region. The variation of electricity produced by the department is a function of the theoretical area. The larger the theoretical area, the greater the potential for electrical energy. It is 0.0009% of the total area of the Western Region. The area covered by PV panels will be reduced if their efficiency is higher. Suri et al. [24], had obtained an average theoretical area of 0.6% for the 25 countries of the European Union. The variation observed between the theoretical areas is related to the electrical energy demand and the solar energy received per square meter in an area. In total, 92,992 households would be electrified through solar electricity. This represents an electricity access rate of 25.08% in the West Cameroon region.

### 4.3 Electrical potential generated by the different biomass sources

The total potential of theoretical electrical energy generated annually by biomass is approximately, 8735 GWh/year and represents 8586.42 GWh/year, 135.53 GWh/year and 13.05 GWh/year for agricultural residues, animal dung and forest residues, respectively. This energy will be produced if all the planned biomass residues (70%) are allocated for this purpose. For, these resources are exploited for cooking, heating and soil fertilization [29]. This energy potential is capable of satisfying 54.59 times the total electricity consumption of the West Cameroon Region in 2014 estimated at 160 GWh/year [34] and 35.95 and 23.67 times the forecasts for the year 2022 and 2030 estimated at 242.96 GWh and 368.92 GWh, respectively. This follows an annual growth rate of electrical energy of 5.36% [39]. This energy is capable of meeting 3.52 and 1.98 times the 2020 and 2030 Cameroon residential needs, estimated at 2481 GWh/year [39] and 4406.16 GWh/year. Mboumboue and Njomo [6], had found that the total potential for electricity production from all sources is estimated at 67,500 GWh/year, which is equivalent to about 12 times the total electricity production of Cameroon in 2010. This high electricity potential shows that the exploitation of this source will not only satisfy the electricity needs of 18,408,853 households in the West Cameroon region and its surroundings, but will also solve load shedding problems and improve access to electricity in this region. The electrical energy potential of biomass is high for agricultural residues followed by animal waste and lower for forest residues with a potential of 8586.42 GWh/year, 135.53 GWh/year and 13.05 GWh/year, respectively. These values show that the potential of agricultural residues represents 98.30% of the estimated biomass energy potential in the Region. This potential represents 6.10% of the electrical energy potential obtained by Mboumboue and Njomo [6]. However, Mboumboue and Njomo [6], in his work, had obtained an estimated energy potential of 80,388.89 GWh/year, 58,055.56 GWh/year, 4331.75 GWh/year and 305.56 GWh/year for agricultural residues, firewood, animal waste and charcoal in Cameroon. The electrical energy potential obtained in the Western Region represents 10.68%, 3.12% and 0.02% of the electrical potential obtained by Mboumboue and Njomo [6], respectively for electrical energy produced by agricultural residues, animal waste and forest residues. This energy potential is the result of 70% of the residues generated in the region. It largely meets the needs of the population of West Cameroon. After analysis, it appears that the recoverable fraction of residues necessary to satisfy the current energy demand (242.96 GWh) of the population is 1.93%. The locality produces a lot of agricultural residues because more than 70% of the active population of the region is employed in the agro-pastoral sector. The observed variation in the energy potential of biomass residues may be subject to errors inherent in data collection, seasonal variations, production levels [40] and related to the impact of COVID-19. This pandemic affected data collection through reduced trade due to state containment measures to reduce the spread of the disease. In addition, environmental considerations also require that stumps and roots not be harvested. They have a soil stabilization function [32].

### 4.4 Household access to electricity

Electricity generated from forest residues and hydropower can only meet the needs of 7.42% and 6.64% of households. However, only 33.40% of residences do not have access to electricity in the Western Region. Solar energy can fill this gap up to 25.08%. However, electrical energy produced by animal and agricultural biomass would meet all the needs of households that do not have access to electricity in the West Cameroon region. For, 37.9% of households do not have access to electricity in Cameroon [34]. Biomass residues will improve the accessibility and reliability of renewable electricity supply in the entire Western region if a thermal power plant is built for this purpose. Studies conducted in 2021 by the National Institute of Statistics (INS) [41] showed that the growth rate in the agricultural production sector is +0.6% in 2020 after 3.9% in 2019. So, there will be enough biomass to operate thermal power plants; provided that they are assigned to the production of energy. The energy produced by biomass will help boost the economy of the West Cameroon region and improve the living conditions of households and the production level of small and medium industries. Better yet, it will increase the level of industrialization of the region while reducing the unemployment rate.

The results obtained show that the production of energy with biomass resources, solar irradiation and the flow of rivers with a drop greater than or equal to 2 m are intermittent and may reduce their reliability. It is therefore better to realize hybrid systems (thermal power plants with cogeneration-solar) adapted in local decentralized structures to overcome this limitation. The Bamboutos and Noun departments provide more of the same agricultural residues for solar energy. Noun also has more than half of the area (7690 km^2^) of the West [42]. It would therefore be necessary for the State to build two hybrid power plants; one at the Bamboutos-Mifi-Menoua border and the other at the Noun-Koung Khi border, to reduce distances and make the transport and accessibility of raw material to the plants more economical. These power plants must be close to Mifi because it has a density of 806 Hab/km^2^ [42] to satisfy compared to the other departments which have density lower than 310 Hab/km^2^ [42]. The combination of these two resources will ensure the stability of the network and the continuity of energy access for the population, while offering an ecological and economical solution. Hybrid energy exploitation leads to reduced maintenance costs, greater operational efficiency, and improved infrastructure reliability. The reduction in the amount of agricultural residues in the off-season can be corrected by forest biomass. Renewable energy resources therefore remain potential with a sustainable solution to capitalize on to improve household living conditions in the West Cameroon region.

The primary materials used to determine the energy potential are common goods and can be used for several activities: irrigation, livestock, mulching, soil fertility restoration, drinking water, hydroelectricity and many others. Indeed, this study shows that there is a link between water-energy-food. It must be evaluated in order to avoid conflicts that could arise when exploiting its resources. According to Garcia and You [43], a Nexus approach can support the transition to a green economy and enable efficient use of resources and greater policy coherence, given the increasing interdependence across sectors in space and time. A reduction in negative economic, social and environmental externalities can increase overall resource use efficiency.

## 5 Conclusion

The aim of this study was to evaluate the potential for hydroelectric, solar and biomass (agricultural, forestry and animal biomass) energy. In total, 48 waterfall points were explored, with an estimated hydroelectric energy potential of 11.68 GWh/year. The solar energy potential obtained on a theoretical surface of 0.0009% of that of each department is estimated at 44.12 GWh/year and that of the different types of biomass is 8586.42 GWh/year, 135.53 GWh/year and 13.05 GWh/year, respectively for agricultural residues, animal waste and forestry in the West Cameroon region. These values show that the potential of the agricultural residues represents 98.3% of the biomass energy potential in the West Cameroon region. These potentials can satisfy the low-voltage consumption needs of 18,095,715, 285,637, 92,992, 27,501, 24,619 households; which represents an electricity access rate of 4881.46%, 77.05%, 25.08%, 7.42% and 6.64%, respectively for agricultural residues, animal dung, solar, forestry residues and hydraulic energy. Energy from agricultural residues satisfies the 33.40% of the population without access to electricity in the Western Region. Biomass resources contributed significantly to the supply and improvement of the energy access rate in the West Cameroon region. In addition to the use of biomass for energy purposes, producers of these residues can also market them to benefit from the economic benefits. These different energy resources will contribute to reducing the poverty level in the West Cameroon region. The energy resource potential characterized in this study makes it possible to develop energy policy design and ensure integrated and sustainable management of renewable energy supply. For effective use of these different energy sources, two hybrid systems (thermal power plant with cogeneration-solar) can be set up to facilitate the exploitation of these resources which will contribute to the best preservation of our environment. Better yet, evaluate the nexus between water-energy-food to avoid the conflicts that could arise in the use of its resources.

## 6 Study limitation and future research

The limitations of this research are the lack of recent and accurate data for biomass residues. Therefore, data from 2018 to 2021 were used for this study. Further work can focus on other renewable energy sources such as biofuels and wind in the region. Better yet, do an economic analysis of hybrid plants to assess the feasibility of the investment.

## Author contribution statement

Nasse FETIO NGOUNE: Conceived and designed the experiments; Performed the experiments; Analyzed and interpreted the data; Contributed reagents, materials, analysis tools or data; Wrote the paper.

Boris Merlain KANOUO DJOUSSE: Conceived and designed the experiments; Analyzed and interpreted the data; Wrote the paper.

Grisseur Henri DJOUKENG: Conceived and designed the experiments; Wrote the paper.

Cherelle Grace F. NGUIMEYA: Performed the experiments; Wrote the paper.

Kewir Julius TANGKA; Martin TCHOFFO: Contributed reagents, materials, analysis tools or data.

## Funding statement

This research did not receive any specific grant from funding agencies in the public, commercial, or not-for-profit sectors.

## Data availability statement

Data will be made available on request.

## Declaration of interest’s statement

The authors declare no conflict of interest.

## Figures and Tables

**Table:** 

Speculation	Residue types	Water content	Residue to produce ratio [RPR]	Energy use factor	Surplus availability factor	Lower heating value [MJ/kg]	Publication
Groundnut	Peanut straw	18.86	1.7	0.07	0.76	17.58	Kemausuor et *al*. 2015 [44]
	Peanut shell		0.323	0	1	11.23	Bhattachaya et *al*. 1989 [45]
Sweet banana	Banana leaves	40	0.35			11.37	Rodriguez et *al*. 2016 [46]
	Banana stem	40	5.6			11.66	Rodriguez et *al*. 2016 [46]
	Leaf sheath	15	0.25			17	Rodriguez et *al*. 2016 [46]
Banana Plantain	Plantain leaves	40	0.35			12.12	Rodriguez et *al*. 2016 [46]
	Leaf sheath	40	4.16			10.85	Rodriguez et *al*. 2016 [46]
	Plantain peels	15	0.25			17	Rodriguez et *al*. 2016 [46]
Cocoa	Cocoa shell	15	1			15.48	Ackom et *al*. 2013 [47]
	Cocoa pod	15	1			15.48	Ackom et *al*. 2013 [47]
Coffee	Coffee pod	15	2.1			12.56	Ackom et *al*. 2013 [47]
Bean	Bean straw	10	1.9			12.38	Asakereh et *al*. 2014 [48]
Yam	Yam peels	15	0.5			10.61	Kemausuor et *al*. 2015 [44]
Corn	Corn stalk	20	2	0	1	12.6	Perera et *al*. 2005 [49]
	Corn husk	8.65	0.25	0.193	0.67	16.63	Black and Veatch, 1999 [50] DEDP. 1992 [51]
	Corn spathe	20	0.9			12,6	UNEP. 2013 [33]
Cassava	Cassava stems	20	1.24	0	0,407	17.5	Kemausuor et *al*. 2015 [44]
	Cassava peelings	20	0.34			13.38	Kemausuor et *al*. 2015 [44]
Oil palm	Palm oil fiber	10.11	0.147	0.858	0,134	16.19	DEDP. 1995 [52]
	Palm oil husk	13	0.049	0.588	0.037	17	DEDP. 1995 [52]
	Palm oil leaves	48,34	2.604	0	1	7.97	DEDP. 1995 [52]
	Empty palm oil bunches	8.81	0.428	0.03	0.584	16.44	DEDP. 1995 [52]
	Male palm oil bunches	13.82	0.233	0	1	14.86	DEDP. 1995 [52]
Sweet potato	Sweet potato peels	15	0.5			10.61	Kemausuor et *al*. 2015 [44]
Potato	Potato peels		1.1			13.6	Koga. 2008 [53]
Rice paddy	Rice husk	8.83	0.23	0.531	0.469	12.85	Sajjakulnukit et *al*. 2005 [54]
	Rice straw	8.17	0.447	0	0.684	8.83	Bhattachaya et *al*. 1989 [45] DEDP. 1992 [51]
Soybean	Soybean straw	15	3.5	0.007	0.76	12.38	Kemausuor et *al*. 2015 [44]
Cocoyam	Cocoyam peels		0.5			14.24	Ezealigo et *al*. 2021 [55]
Tomato	Tomato stems	8	0.3			19.5	Jeguirim et *al*. 2012 [56]
	Tomato leaves	8	0.3			19.5	Jeguirim et *al*. 2012 [56]

**Table:** 

N°	Village	Neighbourhood	Watercourse	K [m]	Q [l/s] 2002	Q [l/s] 2022	Pel [kWh]
Department of Bamboutos							
1	Babadjou	Tounga	Douvo’o	140		1.19	31.38
2	Bamessingué	Bamessingué	Bamesso	27		4.00	20.33
3	Bamessingué	Bamendou	Sa’afim	9		5.59	9.48
4	Bangang	Mepibea	Mepibea	35		25.87	170.54
5	Bangang	Bantsiet	Kououloh	2.06		11.05	4.29
6	Bangang	Bantsiet	Twolepi	15		12.86	36.34
7	Bangang	Troumepea	Mba’a	15		10.74	30.34
8	Bangang	Baladjeutsa	Tchouotoum	30		12.27	69.34
Department of Haut Nkam							
9	Bafang	Mouenkeu	Mouenkeu	36		398.58	2702.64
10	Bakoven	Make	Ngoum	5	180	172.20	162.17
Department of Hauts Plateaux							
11	Baham	Chengne	Vac-Vac	43		3.30	26.69
12	Baham	Gogoua	Kanmalin	7		5.53	7.29
Department of Menoua							
13	Bafou-Nord	Mintsi	Mintsi	83	80	0.46	7.21
14	Bamendou	Balefock	Folepé	34	800	303.88	1946.06
15	Fongo-Tongo	Apouh	Mami water	110	50	20.29	420.40
16	Fongo-Tongo	Toutchouet	Toussa	43	60	4.34	35.15
17	Fongo-Tongo	Alloh	Talla	96	150	263.76	4769.16
18	Fongo-Tongo	Lefok	Sentse	41	120	26.90	207.69
19	Fongo-Tongo	Fossong	Folepeh	21	700	93.94	371.57
20	Fongo-Tongo	Yaguem	Folefok	12	800	11.48	25.96
21	Fongo-Tongo	Loung	Matsoung	24	950	39.01	176.36
22	Fongo-Tongo	Meloung	Fotoutcheut	84		2.61	41.22
23	Foreké	Migwnog	Lefock	18	120	283.49	961.12
24	Foreké	Falaise pont1	Falaise	55		54.86	568.26
25	Foreké	Falaise pont2	Meguimme	29		35.44	193.59
26	Foto	Fiala’a	Foka	50		71.43	672.69
27	Foto	Lingang	Lingang	40		123.32	929.10
28	Foto	Tsinkop	Setsa	44	40	3.21	26.63
29	Foto	Lepeh	Tchoulepeh	55	280	25.23	261.35
30	Foto	Tsinkop	Dschang water	43		140.01	1133.94
Department of Mifi							
31	Badeng	Mifi	Mifi	20	400	1320.32	4973.69
32	Baleng	Tougoum	Sinte	100	10	2.44	45.89
33	Baleng	Nefolom	Manema	20	400	74.71	281.42
34	Baleng	Nkoujeh	Nefelom	22		19.41	80.45
35	Baleng	Famleng	Magnekie	10	300	177.57	334.46
36	Baleng	Bapi	Bapi	33		67.64	420.41
37	Bamougoum	Metchié	Metchié	20	750	1080.46	4070.14
Department of Ndé							
38	Bangoua	Bangoua	Tahmveue	24		289.88	1310.40
39	Bangoulap	Fantchouet	Baba’a 1	15		6.86	19.39
40	Bangoulap	Fantchouet	Baba’a 2	17		22.74	72.82
Department of Nkoung-kih							
41	Bandjoun	Dja	Rhèshior	11		118.65	245.82
Department of Noun							
42	Foumban	Njiloum	Nchi	20		243.45	917.08
43	Foumban	Njintout (plateau)	Nchi (plateaux)	10		233.28	439.39
44	Kouoptamo	Choutjot	Njot	15		341.32	964.32
45	Koutaba	Koundja	Ndja	22		190.71	790.25
46	Koutaba	Kouchankap	Ndja (koutaba)	11		185.65	384.64
47	Koutaba	Ngoundoup	Ndoup	10		109.82	206.84
48	Mansouen	Feyouom	Moum	9		253.15	429.13
**TOTAL**						**32004.85**	

## APPENDIX

Appendix 1. RPRi, LVHi, SAF, EUF parameters for each agricultural residue.

* DEDP: Department of Energy Development and Promotion; UNEP: United Nations Development Programme.

## Appendix 2

Hydraulic characteristics of rivers with falls and stunts

*Pel: electrical energy at the output of the alternator in kWh; Q: water flow rate in l/s and K Gross head of water in m.
